# Accuracy of the Brief Cognitive Screening Battery for diagnosing Alzheimer's disease defined by cerebrospinal fluid biomarkers and AT(N) classification: a case-control study

**DOI:** 10.1590/0004-282X-ANP-2021-0012

**Published:** 2021-11-30

**Authors:** Patrícia Regina Henrique Peles, Larissa de Souza Salvador, Leonardo Cruz de Souza, Paulo Caramelli

**Affiliations:** 1 Universidade Federal de Minas Gerais, Programa de Pós-Graduação em Neurociências, Belo Horizonte MG, Brazil. Universidade Federal de Minas Gerais Programa de Pós-Graduação em Neurociências Belo Horizonte MG Brazil; 2 Universidade Federal de Minas Gerais, Faculdade de Medicina, Departamento de Clínica Médica, Grupo de Pesquisa em Neurologia Cognitiva e do Comportamento, Belo Horizonte MG, Brazil. Universidade Federal de Minas Gerais Faculdade de Medicina Departamento de Clínica Médica Belo Horizonte MG Brazil; 3 Universidade Federal de Minas Gerais, Faculdade de Medicina, Programa de Pós-Graduação em Ciências Aplicadas à Saúde do Adulto, Belo Horizonte MG, Brazil. Universidade Federal de Minas Gerais Faculdade de Medicina Programa de Pós-Graduação em Ciências Aplicadas à Saúde do Adulto Belo Horizonte MG Brazil

**Keywords:** Alzheimer Disease, Cognitive Dysfunction, Diagnosis, Biomarkers, Cognition, Doença de Alzheimer, Disfunção Cognitiva, Diagnóstico, Biomarcadores, Cognição

## Abstract

**Background:**

Validation of cognitive instruments for detection of Alzheimer's disease (AD) based on correlation with diagnostic biomarkers allows more reliable identification of the disease.

**Objectives:**

To investigate the accuracy of the Brief Cognitive Screening Battery (BCSB) in the differential diagnosis between AD, non-AD cognitive impairment (both defined by cerebrospinal fluid [CSF] biomarkers) and healthy cognition, and to correlate CSF biomarker results with cognitive performance.

**Methods:**

Overall, 117 individuals were evaluated: 45 patients with mild cognitive impairment (MCI) or mild dementia within the AD continuum defined by the AT(N) classification [A+T+/-(N)+/]; 27 non-AD patients with MCI or mild dementia [A-T+/-(N)+/-]; and 45 cognitively healthy individuals without CSF biomarker results. All participants underwent evaluation using the BCSB.

**Results:**

The total BCSB and delayed recall (DR) scores of the BCSB memory test showed high diagnostic accuracy, as indicated by areas under the ROC curve (AUC): 0.89 and 0.87, respectively, for discrimination between AD and non-AD versus cognitively healthy controls. Similarly, total BCSB and DR displayed high accuracy (AUC-ROC curves of 0.89 and 0.91, respectively) for differentiation between AD and controls. BCSB tests displayed low accuracy for differentiation between AD and non-AD. The CSF levels of biomarkers correlated significantly, though weakly, with DR.

**Conclusions:**

Total BCSB and DR scores presented good accuracy for differentiation between patients with a biological AD diagnosis and cognitively healthy individuals, but low accuracy for differentiating AD from non-AD patients.

## RESUMO

**Antecedentes:**

A validação de testes cognitivos para identificação da doença de Alzheimer (DA) definida por biomarcadores aumenta a confiabilidade diagnóstica.

**Objetivos:**

Investigar a acurácia da Bateria Breve de Rastreio Cognitivo (BBRC) no diagnóstico diferencial entre DA, comprometimento cognitivo não-DA (ambos diagnósticos definidos por biomarcadores no líquido cefalorraquidiano-LCR) e indivíduos cognitivamente saudáveis, e investigar correlações entre desempenho nos testes e concentrações dos biomarcadores no LCR.

**Métodos:**

No total, 117 indivíduos foram avaliados. Quarenta e cinco pacientes com comprometimento cognitivo leve (CCL) ou demência leve com diagnóstico do continuum de DA definido pela classificação AT(N) [A+T+/-(N)+/-], 27 pacientes com CCL ou demência leve não-DA [A-T+/-(N)+/-], e 45 controles cognitivamente saudáveis sem estudo de biomarcadores no LCR. Os participantes foram submetidos à BBRC.

**Resultados:**

O escore total da BBRC e a evocação tardia (ET) no teste de memória da BBRC apresentaram elevada acurácia diagnóstica na diferenciação entre DA e não-DA versus controles, indicada pelas áreas sob a curva ROC (AUC) de 0,89 e 0,87, respectivamente. De modo semelhante, o escore total da BBRC e a ET mostraram elevadas acurácias (AUC-ROC de 0,89 e 0,91, respectivamente) para o diagnóstico diferencial entre DA e controles. A acurácia da BBRC foi baixa na diferenciação entre DA e não-DA. Os níveis dos biomarcadores no LCR se correlacionaram de forma significativa, embora fraca, com ET.

**Conclusões:**

Os escores totais da BCSB e a ET apresentaram boa acurácia na diferenciação entre pacientes com diagnóstico biológico de DA e controles cognitivamente saudáveis, mas baixa acurácia para diferenciar DA de não-DA.

## INTRODUCTION

Alzheimer's disease (AD) is the leading cause of dementia worldwide^1^^,^^2^^,^^3^, although often underreported^4^^,^^5^^,^^6^. Until recently, AD was diagnosed based solely on identification of a characteristic cognitive profile and through ruling out other diseases using ancillary tests. Lately, important advances have been achieved through development of specific biomarkers^7^. 

The cerebrospinal fluid (CSF) biomarker profiles associated with AD consist of reduced concentration of beta-amyloid (Aβ42) and increased concentrations of total tau (T-Tau) and phosphorylated tau (P-Tau). Detection of these biomarkers, by means of CSF analysis or neuroimaging methods, allows a biological diagnosis of AD and differentiation from non-AD dementias through the AT(N) classification. In the AT(N) system, A+ individuals, regardless of whether T and (N) are + or -, are qualified as presenting the *continuum* of the AD pathological process. However, determining these diagnostic biomarkers is costly or invasive, besides being commonly unavailable. Thus, the most-used diagnostic methods are clinical assessment, laboratory tests and structural neuroimaging^8^.

The Brief Cognitive Screening Battery (BCSB) is a useful tool for detect dementia, particularly AD^9^. Several studies have investigated the psychometric characteristics of the BCSB^10^^,^^11^^,^^12^. However, the BCSB has not been investigated or validated among patients with a biological AD diagnosis, which could enhance the evidence for its clinical use. 

The present study aimed to investigate the BCSB for diagnosing the AD *continuum* and the association between BCSB scores and CSF biomarker concentrations.

## METHODS

Our institution’s research ethics committee approved the study.

### Participants

The sample was divided into AD (i.e., AD *continuum*), non-AD and control groups. Individuals with schooling levels of less than four years or with scores below 20 points in the Mini-Mental State Examination (MMSE)^13^^,^^14^ were excluded. 

AD and non-AD patients presented a clinical diagnosis of mild cognitive impairment (MCI) or mild dementia. All patients underwent CSF biomarker analysis, with concentration measurements on Aβ42, T-tau and P-tau. The diagnostic categorization of AD and non-AD was purely biological, independent of the cognitive results. Thus, two diagnostic classifications were established: 1) clinical, in accordance with consensual criteria for AD^15^^,^^16^ behavioral variant frontotemporal dementia^17^, vascular dementia^18^^,^^19^, primary progressive aphasia^20^ and dementia with Lewy bodies^21^^,^^22^; and 2) biological, based on CSF biomarkers and on the AT(N) classification. The clinical and biological classifications were performed by independent researchers. 

The cognitively healthy controls used in this study did not have any history of neurological or psychiatric disorders, or depression according to clinical assessment, were not taking medications with cognitive effects and presented normal MMSE^13^^,^^14^ scores for their age and education^23^. CSF biomarkers were not available for controls.

### Instruments

The participants underwent MMSE and BCSB assessments. The BCSB comprises three tests: 1. Figure memory test (FMT)^24^, including naming, incidental memory, immediate memory, learning, delayed recall (DR) and recognition; 2. Verbal fluency (VF) test, in animals/minute^25^ 3. Clock drawing test (CDT)^26^. 

In the FMT, a board with 10 drawings is presented to participants, who are asked to name them; then, without the board, these subjects are asked to evoke the drawings (incidental memory). Subsequently, the board is shown twice for 30 seconds, for two recalls (immediate memory and learning). VF and CDT are administered as interference tests, followed by DR of the drawings and recognition. BCSB administration usually takes eight to 10 minutes.

Total scores were calculated for each task separately and were transformed into z scores based on BCSB^11^ normative data, stratified according to age and education.

### Biological analysis

CSF analyses were conducted in two laboratories, following the same procedures. CSF samples were centrifuged at 3,000 revolutions per minute for 10 minutes, at 4ºC, no more than four hours after collection. CSF aliquots were frozen in polypropylene tubes at -80ºC until analysis. Biomarkers were measured by means of the enzyme-linked immunosorbent assay (ELISA) technique using INNOTEST hTAU Ag, PHOSPHO-TAU (181P) and β-Amyloid (1-42) kits (Fujirebio Europe NV, Gent, Belgium), following the manufacturer’s instructions. The reference values for AD diagnosis were Aβ42 < 700 pg/mL, T-tau > 375 pg/mL and P-tau > 60 pg/mL. The reference values for non-AD diagnoses were Aβ42 ≥ 700 pg/mL, T-tau ≤ 375 pg/mL, P-tau ≤ 60 pg/mL^27^.

### Statistical analysis

First, we conducted descriptive analysis on the sociodemographic data and on the raw scores from the cognitive tests. Then, we used the Kruskal-Wallis test with z-scores controlled according to age and education, to investigate differences in BCSB subtests between AD *vs*. non-AD *vs*. controls. Effect sizes were calculated. The Kendall method, with Bonferroni corrections, was used to explore correlations between biomarkers and cognitive performance. The sensitivity and specificity of BCSB subtests for diagnosing clinical groups were determined through receiver operating characteristic (ROC) curves. Lastly, logistic regression analysis was used to investigate the likelihood of identifying clinical cases using BCSB subtests. 

## RESULTS

The AD group included 45 participants (57.7% women), with a mean age of 65.3 years (SD = 6.5) and mean schooling of 13.1 years (SD = 5.1) [34 A+T+(N)+; 2 A+T+(N)-; 9 A+T-(N)-]. The AD patients had a mean symptom duration of 2.7 years (SD = 1.8). The non-AD group included 27 participants (37.0% women), with a mean age of 64.5 years (SD = 6.4) and mean schooling of 11.9 years (SD = 4.6) [21 A-T-(N)-; 1 A-T-(N)+; 1 A-T+(N)-; 4 A-T+(N)+]. The non-AD group included 13 participants with behavioral variant temporal dementia, eight patients with MCI, three with semantic variant-primary progressive aphasia, one with vascular dementia, one with dementia with Lewy bodies and one with dementia of undefined etiology. The non-AD patients had a mean symptom duration of 2.1 years (SD = 1.1). The control group included 45 participants (44.4% women), with a mean age of 68.9 years (SD = 5.6) and mean schooling of 10.0 years (SD = 5.1). Table 1 presents sociodemographic and cognitive performance data for each group. 

**Table 1.** Sociodemographic and cognitive data of the AD, non-AD and control groups.

Subtests	AD (n = 45)	Non-AD (n = 27)	Controls (n = 45)	K	P	Post-hoc (Dunn test)	Effect size
Age	65.3 (6.5)	64.5 (6.4)	68.9 (5.6)	5.35	< 0.005	0 = 1 < 2	d = 0.31
Schooling	13.1 (5.1)	11.9 (4.6)	10.0 (5.1)	4.48	< 0.01	0 = 1 > 2	d = 0.28
Sex					p < 0.007	x² = 5.28	
Men	19	17	16				
Woman	26	10	29				
MMSE	24.1 (2.8)	24.2 (2.1)	28.0 (1.2)	14.31	< 0.001	0 = 1 < 2	*η* ^*2*^ = 0.10
Naming	9.8 (0.5)	9.7 (0.8)	9.9 (0.2)	0.59	0.74	-	-
Inc. Mem	4.5 (1.9)	5.1 (1.8)	5.8 (1.3)	9.34	< 0.001	0 < 2; 1 = 2	*η* ^*2*^ = 0.06
Im. Mem	6.2 (1.64)	6.7 (1.6)	8.1 (1.19)	31.77	< 0.001	0 = 1 < 2	*η* ^*2*^ = 0.25
Learning	7.0 (1.7)	7.4 (2.2)	8.9 (1.0)	26.26	< 0,001	0 = 1 < 2	*η* ^*2*^ = 0.21
DR	4.5 (2.2)	6.1 (2.4)	8.3 (1.2)	48.46	< 0.001	0 < 1 < 2	*η* ^*2*^ = 0.40
Recognition	8.7 (2.0)	8.0 (2.2)	9.8 (0.4)	16.26	< 0.001	0 = 1 < 2	*η* ^*2*^ = 0.12
VF	13.3 (5.1)	11.4 (4.6)	17.7 (4.7)	25.61	< 0.001	1 < 0 < 2	*η* ^*2*^ = 0.21
CDT	6.9 (2.3)	6.6 (2.3)	8.3 (1.7)	6.48	< 0.03	-	-
BCSB total	59.4 (12.4)	59.7 (10.7)	77.1 (7.4)	31.98	< 0.001	0 = 1 < 2	*η* ^*2*^ = 0.26

0: AD; 1: non-AD; 2: Control; MMSE: Mini-Mental State Examination; Inc. Mem: incidental memory; Im. Mem: immediate memory; DR: delayed recall; VF: verbal fluency; CDT: clock drawing test; BCSB: Brief Cognitive Screening Battery.

The AD patients performed significantly worse than both the non-AD patients and the controls only in the DR subtest. In the incidental memory subtest, the AD patients displayed significantly lower performance than the controls, but performed similarly to the non-AD patients. In the VF subtest, the AD patients performed better than the non-AD participants, but worse than the controls. In immediate memory, learning and recognition, the AD and non-AD groups performed significantly worse than the controls, although AD and non-AD performances were similar.

**Figure 1.**
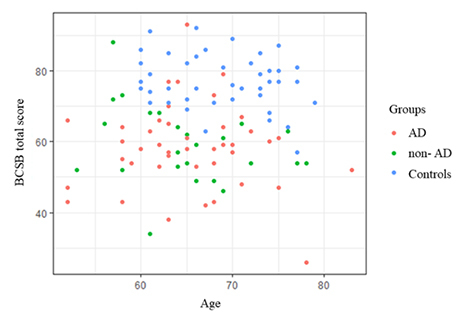
Dispersion according to age and total BCSB score.

Regarding BCSB total scores, the AD and non-AD groups displayed significantly lower performance than the controls. Figure 1 shows the dispersion of cases according to age, total BCSB score and group. 

BCSB: Brief Cognitive Screening Battery.

The area under the ROC curve (AUC), confidence interval, sensitivity, specificity and best cutoff scores were calculated for each BCSB variable for differential diagnoses between AD, non-AD and controls (Table 2). 

**Table 2.** Data from ROC curves for comparisons between clinical groups (AD and non-AD) and controls, and between AD and non-AD patients.

	Variable	Naming	Inc Mem	Im Mem	Learning	DR	Recognition	VF	CDT	BCSB total
Clinical groups vs. Controls	AUC	0.53	0.68	0.79	0.78	0.87	0.69	0.78	0.69	0.89
	95% CI	0.43 to 0.62	0.58 to 0.76	0.71 to 0.86	0.69 to 0.851	0.79 to 0.92	0.59 to 0.77	0.69 to 0.85	0.59 to 0.78	0.83 to 0.95
	Sensitivity	5.56	65.28	51.39	76.39	80.56	48.61	68.18	63.33	88.89
	Specificity	100	64.44	93.33	71.11	77.78	86.67	80.00	75.56	81.94
	Cutoff	≤ 8	≤ 5	≤ 6	< 8	< 7	≤ 9	≤ 13	≤ 8	≤ 68
AD vs. Controls	AUC	0.53	0.71	0.82	0.82	0.91	0.70	0.75	0.67	0.89
	95% CI	0.43 to 0.62	0.60 to 0.81	0.73 to 0.89	0.72 to 0.89	0.84 to 0.96	0.60 to 0.80	0.64 to 0.83	0.56 to 0.77	0.81 to 0.94
	Sensitivity	5.57	71.11	57.78	82.22	68.89	51.11	67.44	62.16	80
	Specificity	100.00	64.44	93.33	71.11	100	86.67	73.33	75.56	91.11
	Cutoff	≤ 8	≤ 5	≤ 6	≤ 8	≤ 5	≤ 9	≤ 14	≤ 8	≤ 67
AD vs. non-AD	AUC	0.51	0.58	0.58	0.60	0.69	0.55	0.63	0.54	0.50
	95% CI	0.39 to 0.63	0.46 to 0.70	0.46 to 0.70	0.48 to 0.71	0.57 to 0.79	0.43 to 0.67	0.50 to 0.75	0.41 to 0.67	0.38 to 0.62
	Sensitivity	100	71.11	57.78	53.33	53.33	26.67	72.09	64.86	80.00
	Specificity	3.7	44.44	59.26	62.96	81.48	85.19	52.17	43.48	7.41
	Cutoff	> 5	≤ 5	≤ 6	≤ 7	≤ 4	≤ 8	> 10	> 5	> 48

AUC: area under the curve; 95% CI: Confidence interval; Criterion: cutoff point; Inc. Mem.: incidental memory; Im. Mem.: immediate memory; DR: delayed recall; VF: verbal fluency; CDT: clock drawing test; BCSB: Brief Cognitive Screening Battery.

**Figure 2.**
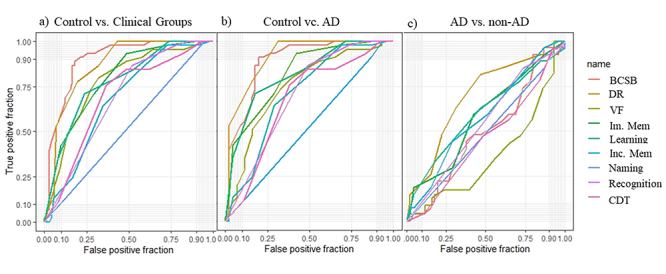
ROC curves for comparisons between groups.

As can be seen in Figure 2 (A, B), naming did not present satisfactory AUC in any of the comparisons. The DR subtest and total BCSB score presented the best AUC values for comparisons between clinical groups and controls, and between AD and controls. None of the BCSB subtests displayed good AUC for differentiation between AD and non-AD (Figure 2C). 

Inc. Mem: incidental memory; Im. Mem: immediate memory; DR: delayed recall; VF: verbal fluency; CDT: clock drawing test; BCSB: Brief Cognitive Screening Battery (total).

In the logistic regression analysis, DR and total BCSB scores displayed the best results regarding diagnostic prediction of clinical groups. The learning subtest of the FMT was the only test that significantly differentiated AD from non-AD cases (Table 3). 

**Table 3.** Results from logistic regression comparisons between groups.

	Variable	Naming	Inc Mem	Im Mem	Learning	DR	Recognition	VF	CDT	BCSB total
Clinical groups *vs*. Controls	Odds ratio	1.00	0.80	1.23	0.99	2.40	1.04	1.17	1.10	1.22
	95% CI	0.82 to 1.21	0.46 to 1.36	0.69 to 2.24	0.58 to 1.68	1.40 to 4.69	0.83 to 1.38	0.94 to 1.46	0.83 to 1.46	1.04 to 1.52
	p value	0.72	< 0.001	< 0.001	0.08	< 0.001	0.18	0.09	0.3	< 0.05
AD *vs*. Controls	Odds ratio	0.9	0.8	1.22	1.23	2.95	0.94	1.06	1.14	1.26
	95% CI	0.63 to 1.23	0.39 to 1.56	0.61 to 2.47	0.59 to 2.14	1.55 to 6.88	0.66 to 1.33	0.75 to 1.37	0.80 to 1.63	1.02 to 1.80
	p value	0.77	< 0.001	< 0.001	< 0.01	< 0.001	0.74	0.58	0.44	0.02
AD *vs*. Non-AD	Odds ratio	0.78	0.71	0.69	1.88	1.38	0.85	0.68	1.01	1.3
	95% CI	0.56 to 1.01	0.31 to 1.50	0.34 to 1.29	1.15 to 3.50	0.90 to 2.38	0.66 to 1.07	0.38 to 1.03	0.67 to 1.57	0.89 to 2.09
	p value	0.2	0.99	0.51	< 0.01	0.09	0.09	0.06	0.92	0.19

Inc. Mem: incidental memory; Im. Mem: immediate memory; DR: delayed recall; VF: verbal fluency; CDT: clock drawing test; BCSB: Brief Cognitive Screening Battery.

Correlations between CSF biomarkers and performance in the BCSB among AD and non-AD patients were weak, but significant between biomarkers and DR. A positive correlation between DR and Aβ42 (K = 0.17; *p* < 0.03), and negative correlations between DR and T-tau (K = -0.24; *p* < 0.003) and P-tau (k = -0.24; *p* < 0.004) were observed. 

## DISCUSSION

The BCSB proved to be a good screening instrument for identifying AD *continuum* and non-AD patients, as defined through the CSF biomarkers and AT(N) classification system, in MCI or mild dementia stages, with good sensitivity and specificity. In most subtests, AD patients performed worse than controls. Moreover, the DR subtest displayed good specificity for differentiating AD from non-AD, although with low sensitivity.

The sensitivity and specificity in our study were lower than those found in previous investigations using the BCSB^12^^,^^13^^,^^16^. It is possible that inclusion of non-amnestic AD patients, together with FTD patients with possible memory changes in the non-AD group, may have decreased BCSB accuracy. Furthermore, the increased diagnostic precision determined by biomarkers may also have influenced the results. It should also be considered that the AT(N) classification does not encompass the full spectrum of possible pathophysiological changes associated with aging. Accordingly, new CSF biomarkers (e.g. neurofilament light chain and neurogranin) have been used to optimize dementia diagnoses^28^. In addition, cognitive deficits are not specific for each clinical condition and usually overlap across different diseases^29^. In sum, our results confirm that cognitive tests are sensitive tools for MCI/dementia screening, but the correspondence between clinical and underlying pathological features is not linear. 

The BCSB displayed good diagnostic accuracy, thus corroborating previous results^11^^,^^12^^,^^13^^,^^30^. DR was the best BCSB subtest, in comparing AD and controls. Previous studies^31^^,^^32^ identified that the BCSB DR test was superior to CERAD DR among illiterate individuals^33^^,^^34^ DR, while these tests had similar accuracy among literate people. 

Interestingly, the learning subtest of FMT was the only significant variable in the logistic regression to discriminate between AD and non-AD. However, DR was only marginally significant, and the results suggest that this test was also able to discriminate between AD and non-AD patients. The ROC curve analysis showed that DR was slightly superior to learning, with similar sensitivity, but with greater specificity. Thus, caution is needed in interpreting these results, because our non-AD group included patients with different etiologies and also with episodic memory deficits.

Negative correlations were found between DR and CSF T-tau and P-tau concentrations, and a positive correlation between DR and Aβ42. However, all these correlations were weak. It is important to highlight that elevated T-tau levels in the CSF, indicative of neurodegeneration or (N+), were observed in 3/4 of AD patients, but in less than 10% of non-AD cases.

Investigation of CSF biomarkers in association with cognitive testing contributes to understanding deficits that may be attributable to the biological substrates of AD. In two studies that investigated CSF biomarkers in relation to cognition, Rolstad et al. observed that Aβ42 levels correlated with episodic memory deficits, starting from the onset of the disease^35^, while T-tau levels had a small to moderate influence on all cognitive domains, except for visuospatial abilities, in patients with MCI^36^. Some studies^37^^,^^38^ correlated biomarkers and cognition in a temporal pattern, such that cognitive performance correlated first with Aβ42, then with T-tau and P-tau. This suggested that combination of neuropsychological assessment with CSF biomarkers is useful for making AD differential diagnoses. Additionally, the concentrations of P-tau have greater specificity for AD diagnosis, showing good discrimination between AD and frontotemporal dementia, since the levels of this biomarker are more associated with cognition in AD and correlate with disease stage^39^.

The BCSB cutoff scores presented in our study indicate high precision in identifying AD and non-AD, since the diagnoses were based on CSF biomarkers. However, the present study was limited by the lack of biomarker data among the controls and by small sample sizes. In addition, although biomarker analyses were carried out using the same diagnostic kit, the tests were conducted in two laboratories, which might have skewed the biomarker measurements.

Combination of less invasive and more accessible tests makes it possible to overcome the financial and structural challenges of the healthcare system, without neglecting diagnostic reliability. In view of the growing demand for differential diagnoses of dementia, it is necessary to use validated instruments to assist in diagnostic investigation. We conclude that the BCSB displays good accuracy for differentiation between patients with a biological diagnosis of AD, non-AD patients and controls, thus confirming its value as a cognitive screening tool for clinical practice.
